# Risk Factors, Prognostic Factors, and Nomograms for Bone Metastasis in Patients with Newly Diagnosed Clear Cell Renal Cell Carcinoma: A Large Population-Based Study

**DOI:** 10.3389/fsurg.2022.877653

**Published:** 2022-04-01

**Authors:** Hongmin Zhou, Sheng Yang, Tiancheng Xie, Longfei Wang, Sen Zhong, Tianyang Sheng, Guoxin Fan, Xiang Liao, Yunfei Xu

**Affiliations:** ^1^ Department of urology, Shanghai Tenth People’s Hospital, Tongji University School of Medicine, Shanghai, China; ^2^ Department of Orthopedics, Shanghai Tenth People’s Hospital, Tongji University School of Medicine, Shanghai, China; ^3^ Spinal Pain Research Institute, Tongji University School of Medicine, Shanghai, China; ^4^ Shanghai Tongji Hospital, Tongji University School of Medicine, Shanghai, China; ^5^ Shanghai East Hospital, Tongji University School of Medicine, Shanghai, China; ^6^ National Key Clinical Pain Medicine of China, Huazhong University of Science and Technology Union Shenzhen Hospital, Shenzhen, China; ^7^ Guangdong Key Laboratory for Biomedical Measurements and Ultrasound Imaging, School of Biomedical Engineering, Shenzhen University Health Science Center, Shenzhen, China; ^8^ Department of Pain Medicine and Shenzhen Municipal Key Laboratory for Pain Medicine, The 6th Affiliated Hospital of Shenzhen University Health Science Center, Shenzhen, China

**Keywords:** clear cell renal cell carcinoma, bone metastasis, SEER, prognostic factors, nomograms

## Abstract

**Background:**

This study aimed to investigate risk factors and prognostic factors in patients with clear cell renal cell carcinoma (ccRCC) with bone metastasis (BM) and establish nomograms to provide a quantitative prediction of the risk of BM and survival probability.

**Methods:**

The clinicopathological characteristics of patients with ccRCC between January 2010 and December 2015 were obtained from the Surveillance, Epidemiology and End Results (SEER) database. Independent factors for BM in ccRCC patients were identified using univariate and multivariate logistic regression analyses. Prognostic factors for predicting cancer-specific death were evaluated using univariate and multivariate analyses based on a competing risk regression model. We then constructed a diagnostic nomogram and a prognostic nomogram. The two nomograms were evaluated using calibration curves, receiver operating characteristic curves, and decision curve analysis.

**Results:**

Our study included 34,659 patients diagnosed with ccRCC in the SEER database, with 1,415 patients who presented with bone metastasis. Risk factors for BM in patients with ccRCC included age, stage T, stage N, brain metastasis, liver metastasis, lung metastasis, tumor size, and laterality. Independent prognostic factors for patients with ccRCC patients with BM were Fuhrman grade, tumor size, T stage, N stage, brain metastases, lung metastasis, and surgery. For the diagnostic nomogram, the area under the curve values in the training and testing cohorts were 0.863 (95% CI, 0.851–0.875) and 0.859 (95% CI, 0.839–0.878), respectively. In the prognostic cohort, the area under the curve values for 1-, 2-, and 3-year cancer-specific survival rates in the training cohort were 0.747, 0.774, and 0.780, respectively, and 0.671, 0.706, and 0.696, respectively, in the testing cohort. Through calibration curves and decision curve analyses, the nomograms displayed excellent performance.

**Conclusions:**

Several factors related to the development and prognosis of BM in patients with ccRCC were identified. The nomograms constructed in this study are expected to become effective and precise tools for clinicians to improve cancer management.

## Introduction

Renal cell carcinoma is one of the most common cancers worldwide, accounting for approximately 5% of male cancers and 3% of female cancers (1). The three most common histological types of renal cell carcinoma are clear cell renal cell carcinoma (ccRCC), papillary renal cell carcinoma (pRCC), and chromophobe renal cell carcinoma (chrRCC), accounting for 80–90%, 10–15%, and 4%–5% of renal cell carcinomas, respectively (2). Compared with pRCC and chrRCC, ccRCC tends to have a worse prognosis (3, 4).

It is estimated that 20–30% of patients with RCC have metastatic disease at the time of initial diagnosis. Bone is one of the most common sites involved, affecting 30% of patients with metastatic disease (5, 6). It is worth noting that the incidence of bone metastasis is higher in patients with ccRCC (7). Approximately 19.7–33.5% of patients with metastatic ccRCC have bone metastases (8). This can cause skeletal-related events, such as pain, hypercalcemia, pathological fractures, and spinal cord compression, leading to severe morbidity (9). It is clinically significant to clarify the predictors of bone metastases (BM) in ccRCC patients because early identification can help optimize treatment and management to reduce skeletal complications (10).

Furthermore, BM is associated with a poor prognosis of ccRCC, and its presence predicts adverse outcomes of angiogenesis inhibitor therapy (11). Although there have been more options for advanced metastatic RCC in recent years, the median overall survival of patients with ccRCC-BM is only 19.4 months (8). Understanding the prognosis of patients with ccRCC-BM is crucial for personalized treatment decisions. Most studies use the Kaplan-Meier method and Cox proportional risk regression to analyze survival, ignoring other competitive events and thus overestimating the probability of cancer-specific death (12, 13). Therefore, it is necessary to consider competitive risk when evaluating the outcomes of patients with ccRCC-BM.

To our knowledge, some population-based studies on bone metastases of renal cell carcinoma have been published. Still, no study has established a predictive model of BM risk and prognosis for ccRCC patients (14–16). Our research used clinical information of ccRCC patients from the SEER cancer database to assess the risk factors for bone metastases. In addition, we evaluated the prognostic factors affecting survival in patients with ccRCC-BM based on competitive risk analysis.

## Materials and Methods

### Identification of Patients

**Figure 1:**
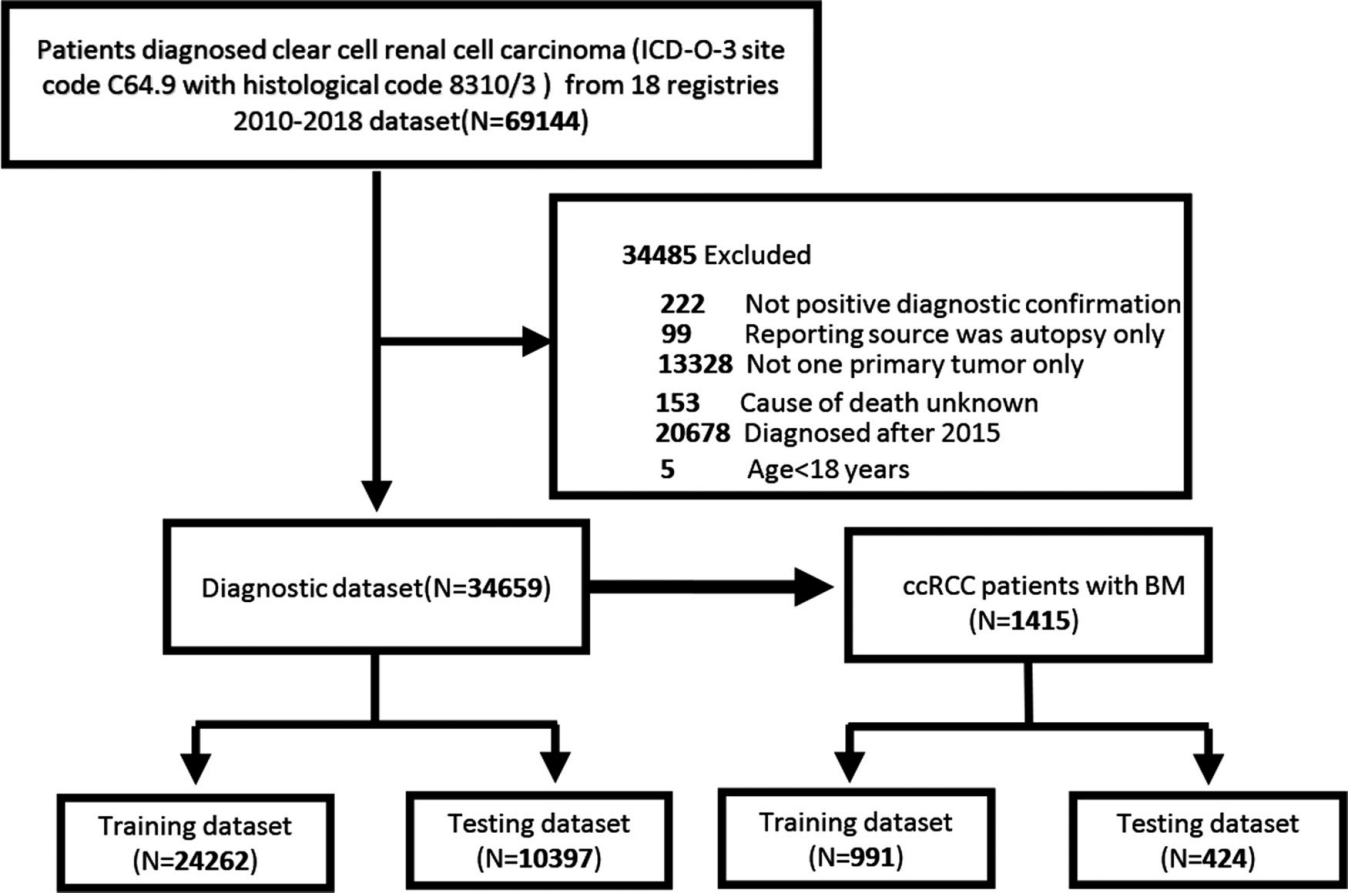
Flowchart of patient selection and model development.

We used SEER*stat software Version 8.3.9 to extract ccRCC (ICD-O-3 site code C64.9 and histological code 8310/3) patient data from the SEER database, which comes from 18 cancer registries and covers approximately 28% of the US population (17). Only patients diagnosed after 2010 were included in our study because information about distant metastasis sites was not recorded in the SEER database until 2010. Furthermore, to record the adequate follow-up time of the patients, we only considered patients diagnosed with ccRCC between 2010 and 2015. In this study, the exclusion criteria for patient selection were age <18 years, more than one primary tumor, not positive diagnostic confirmation; reporting source was autopsy only; cause of death unknown, and cases diagnosed after 2016. Ethic review and informed consent were waived since patients’ information in the SEER database were anonymous and deidentified. The flowchart for patient recruitment is shown in **Figure 1**.

### Study Variables

Clinical information about the patient was extracted from the SEER database. Included sex, race, age at diagnosis, year of diagnosis, marital status, Fuhrman grade, tumor size, tumor laterality, SEER historical stage A, AJCC 7th edition T stage, AJCC 7th edition N stage, surgery administration, lymph node removal, radiation therapy and chemotherapy, invasion beyond capsule, and brain/liver/lung metastasis. In this study, we used X-tile (http://www.tissuearray.org/rimmlab/) to determine the best cutoff values for age based on the cancer-specific mortality rate ccRCC-BM patients. The optimal age cutoff points were 52 and 75 years of age. We then divided the patients into three age groups, 18–52 years old, 53–75 years old, and ≥76 years. The tumor size was categorized into ≤4 cm, 4–7 cm, 7–10 cm, and ≥10 cm. The SEER historic stage includes localized, regional, and distant.

### Statistical Analysis

Patients were randomly assigned to either a training set (70%) or a testing set (30%) for each cohort. The construction of the nomogram was based on the training cohort, and the testing cohort was used to validate the nomogram further. Categorical variables were analyzed using the chi-square test. Univariate and multivariate logistic regression analyses were used to identify clinical risk factors for BM in patients with ccRCC. Based on multivariate logistic regression analysis, we constructed a diagnostic nomogram. To analyze the variables affecting the cancer-specific survival (CSS) of patients with ccRCC-BM, we performed multivariate competing risk analyzes using the cumulative incidence function (CIF), the Fine and Gray test and the proportional subdistribution hazard regression approach. Independent risk factors identified in the competing risk regression analysis were used to construct a nomogram to predict CSS. The identified independent variables were sorted to output relative importance in the final model. The model’s performance was evaluated by plotting receiver operating characteristic (ROC) curves and calibration curves. Decision curve analysis was performed to confirm the clinical application value of the nomogram by calculating net benefits at different threshold probabilities.

SPSS statistical Version 23.0 (IBM, Armonk, NY, USA) and R software (Version 4.0.3) were used for statistical analysis. Missing values less than 60% were handled by the multiple imputation method based on the “mice” package of R. Two-sided *P* values <0.05 were considered statistically significant in analyses.

## Results

### Basic Characteristics of Patients

A total of 34,659 patients diagnosed with ccRCC between 2010 and 2015 from the SEER database were included in our study. We randomly divided them into two datasets, with 24,262 in the training dataset and 10,397 in the testing dataset. Overall, most patients were male (62.5%), and the race of 29,528 patients (85.2%) was white. Most patients were married (64.6%). The highest proportions of the T and N stages were T1 (64.6%) and N0 (94.6%), respectively. The most common Fuhrman grade was grade II (52.6%). In 75.1% of the patients, the tumor size did not exceed 7 cm. Other baseline clinicopathological features of the patients are presented in **Table 1**. The chi-square test indicated that the differences in all variables were not significant between the two cohorts (*P* > 0.05) (Supplementary Table S1). Baseline characteristics of ccRCC patients and ccRCC-BM patients before and after imputation were summarized in Supplementary Tables S2 and S3. Missing data of ccRCC patients and ccRCC-BM patients were described in Supplementary Tables S4 and S5.

**Table 1:** Basic characteristics of patients diagnosed as clear cell renal cell carcinoma.

Variables	Overall (*N* = 34,659)	Without BM (*N* = 33,244)	With BM (*N* = 1,415)				*P*
			Alive (*N* = 192)	Death of ccRCC (*N* = 1,142)	Death of other cause (*N* = 81)	*P*	
Survival.months (mean (SD))	56.69 (27.70)	58.21 (26.88)	54.71 (24.48)	15.39 (16.50)	19.59 (17.94)	<0.001	<0.001
Year.of.diagnosis (%)							
2010	5,033 (14.5)	4,818 (14.5)	18 (9.4)	187 (16.4)	10 (12.3)	<0.001	0.618
2011	5,223 (15.1)	5,013 (15.1)	9 (4.7)	190 (16.6)	11 (13.6)		
2012	5,650 (16.3)	5,407 (16.3)	22 (11.5)	205 (18.0)	16 (19.8)		
2013	5,876 (17.0)	5,632 (16.9)	37 (19.3)	191 (16.7)	16 (19.8)		
2014	6,208 (17.9)	5,953 (17.9)	49 (25.5)	192 (16.8)	14 (17.3)		
2015	6,669 (19.2)	6,421 (19.3)	57 (29.7)	177 (15.5)	14 (17.3)		
Age (%)							
≥76	3,705 (10.7)	3,502 (10.5)	16 (8.3)	170 (14.9)	17 (21.0)	0.022	<0.001
18–52	9,020 (26.0)	8,774 (26.4)	39 (20.3)	199 (17.4)	8 (9.9)		
53–75	21,934 (63.3)	20,968 (63.1)	137 (71.4)	773 (67.7)	56 (69.1)		
Gender (%)							
Female	13,007 (37.5)	12,599 (37.9)	55 (28.6)	330 (28.9)	23 (28.4)	0.993	<0.001
Male	21,652 (62.5)	20,645 (62.1)	137 (71.4)	812 (71.1)	58 (71.6)		
Race (%)							
Black	2,525 (7.3)	2,414 (7.3)	13 (6.8)	88 (7.7)	10 (12.3)	0.59	0.299
Other	2,606 (7.5)	2,513 (7.6)	13 (6.8)	74 (6.5)	6 (7.4)		
White	29,528 (85.2)	28,317 (85.2)	166 (86.5)	980 (85.8)	65 (80.2)		
Marital.status (%)							
No	12,271 (35.4)	11,738 (35.3)	57 (29.7)	440 (38.5)	37 (45.7)	0.021	0.074
Yes	22,388 (64.6)	21,506 (64.7)	135 (70.3)	702 (61.5)	44 (54.3)		
Laterality (%)							
Left	16,951 (48.9)	16,267 (48.9)	101 (52.6)	546 (47.8)	37 (45.7)	<0.001	<0.001
Other	121 (0.3)	67 (0.2)	2 (1.0)	42 (3.7)	10 (12.3)		
Right	17,587 (50.7)	16,910 (50.9)	89 (46.4)	554 (48.5)	34 (42.0)		
Stage (%)							
Distant	3,712 (10.7)	2,297 (6.9)					<0.001
Localized	24,907 (71.9)	24,907 (74.9)					
Regional	6,040 (17.4)	6,040 (18.2)					
Fuhrman.grade (%)							
I	4,012 (11.6)	3,890 (11.7)	19 (9.9)	126 (11.0)	4 (4.9)	0.004	<0.001
II	18,220 (52.6)	17,724 (53.3)	67 (34.9)	360 (31.5)	44 (54.3)		
III	9,859 (28.4)	9,317 (28.0)	74 (38.5)	441 (38.6)	24 (29.6)		
IV	2,568 (7.4)	2,313 (7.0)	32 (16.7)	215 (18.8)	9 (11.1)		
T.Stage (%)							
T1	22,379 (64.6)	22,029 (66.3)	63 (32.8)	258 (22.6)	29 (35.8)	<0.001	<0.001
T2	3,625 (10.5)	3,378 (10.2)	35 (18.2)	200 (17.5)	12 (14.8)		
T3	7,368 (21.3)	6,885 (20.7)	77 (40.1)	388 (34.0)	18 (22.2)		
T4	614 (1.8)	488 (1.5)	7 (3.6)	116 (10.2)	3 (3.7)		
TX	673 (1.9)	464 (1.4)	10 (5.2)	180 (15.8)	19 (23.5)		
N.Stage (%)							
N0	32,774 (94.6)	31,859 (95.8)	150 (78.1)	717 (62.8)	48 (59.3)	<0.001	<0.001
N1	1,285 (3.7)	948 (2.9)	34 (17.7)	284 (24.9)	19 (23.5)		
NX	600 (1.7)	437 (1.3)	8 (4.2)	141 (12.3)	14 (17.3)		
Tumor.size (%)							
≤4	15,773 (45.5)	15,573 (46.8)	31 (16.1)	133 (11.6)	21 (25.9)	<0.001	<0.001
4–7	10,271 (29.6)	9,820 (29.5)	71 (37.0)	344 (30.1)	27 (33.3)		
7–10	5,258 (15.2)	4,829 (14.5)	54 (28.1)	375 (32.8)	16 (19.8)		
≥10	3,357 (9.7)	3,022 (9.1)	36 (18.8)	290 (25.4)	17 (21.0)		
Invasion.beyond.capsule (%)							
Lateral and Medial invasion	773 (2.2)	684 (2.1)	22 (11.5)	171 (15.0)	12 (14.8)	0.113	<0.001
Lateral invasion	2,445 (7.1)	2,219 (6.7)	29 (15.1)	234 (20.5)	11 (13.6)		
Medial invasion	1,774 (5.1)	1,635 (4.9)	20 (10.4)	144 (12.6)	12 (14.8)		
Not present	29,667 (85.6)	28,706 (86.3)	121 (63.0)	593 (51.9)	46 (56.8)		
Brain.metastasis (%)							
No	34,207 (98.7)	32,951 (99.1)	179 (93.2)	997 (87.3)	78 (96.3)	0.005	<0.001
Yes	452 (1.3)	293 (0.9)	13 (6.8)	145 (12.7)	3 (3.7)		
Liver.metastasis (%)							
No	34,085 (98.3)	32,867 (98.9)	180 (93.8)	963 (84.3)	72 (88.9)	0.002	<0.001
Yes	574 (1.7)	377 (1.1)	12 (6.2)	179 (15.7)	9 (11.1)		
Lung.metastasis (%)							
No	32,372 (93.4)	31,617 (95.1)	148 (77.1)	560 (49.0)	50 (61.7)	<0.001	<0.001
Yes	2,287 (6.6)	1,627 (4.9)	44 (22.9)	582 (51.0)	31 (38.3)		
Surgery (%)							
None	2,337 (6.7)	1,549 (4.7)	45 (23.4)	696 (60.9)	47 (58.0)	<0.001	<0.001
Local tumor excision/destruction	1,372 (4.0)	1,362 (4.1)	1 (0.5)	8 (0.7)	1 (1.2)		
Partial nephrectomy	11,497 (33.2)	11,459 (34.5)	14 (7.3)	23 (2.0)	1 (1.2)		
Radical nephrectomy	19,220 (55.5)	18,652 (56.1)	131 (68.2)	405 (35.5)	32 (39.5)		
Nephrectomy, NOS	161 (0.5)	153 (0.5)	1 (0.5)	7 (0.6)	0 (0.0)		
Surgery, NOS	72 (0.2)	69 (0.2)	0 (0.0)	3 (0.3)	0 (0.0)		
Lymph.node.removal (%)							
No	30,651 (88.4)	29,426 (88.5)	154 (80.2)	995 (87.1)	76 (93.8)	0.005	0.028
Yes	4,008 (11.6)	3,818 (11.5)	38 (19.8)	147 (12.9)	5 (6.2)		
Radiotherapy (%)							
No	33,352 (96.2)	32,801 (98.7)	91 (47.4)	422 (37.0)	38 (46.9)	0.007	<0.001
Yes	1,307 (3.8)	443 (1.3)	101 (52.6)	720 (63.0)	43 (53.1)		
Chemotherapy (%)							
No	32,038 (92.4)	31,463 (94.6)	86 (44.8)	450 (39.4)	39 (48.1)	0.136	<0.001
Yes	2,621 (7.6)	1,781 (5.4)	106 (55.2)	692 (60.6)	42 (51.9)		

### Risk Factors for Bone Metastasis in ccRCC Patients and Relative Importance

**Figure 2:**
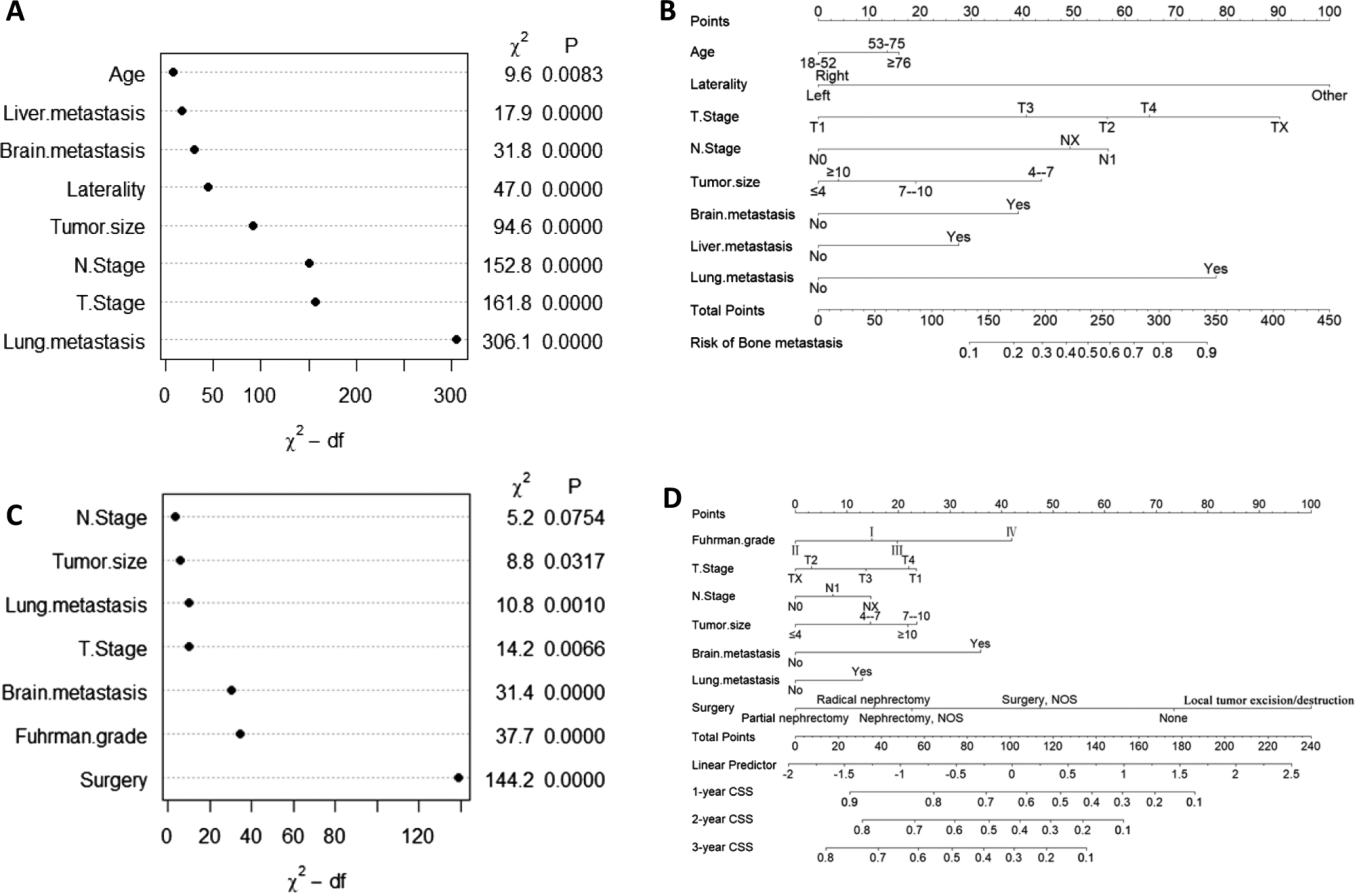
Variable importance and nomogram in the diagnostic cohort (**A** and **B**) and in the prognostic cohort (**C** and **D**).

Univariate logistic regression analysis showed that 11 predictors were associated with BM in ccRCC patients, including age, sex, laterality, Fuhrman grade, T stage, N stage, tumor size, invasion beyond the capsule, brain metastasis, liver metastasis, and lung metastasis (Supplementary Table S6). We used these predictors to perform the multivariate logistic regression analysis. The results showed that age, T stage, N stage, laterality, tumor size, brain metastasis, liver metastasis, and lung metastasis were independent predictors of BM in patients with ccRCC (**Table 2**). Lung metastasis was identified as the most critical risk factor, followed by T stage, N stage, and tumor size (**Figure 2A**).

**Table 2:** Multivariate logistic regression analysis of BM in ccRCC patients and Multivariate competing risk analysis in ccRCC patients with BM.

Variables	Logistic			Competing risk		
	OR	95% CI	*P*	SHR	95% CI	*P*
Age						
≥76	Ref			Ref		
18–52	0.71	0.54–0.92	0.01	0.83	0.64–1.08	0.16
53–75	0.94	0.76–1.17	0.575	0.89	0.72–1.11	0.32
Gender						
Female	Ref					
Male	1.15	0.98–1.34	0.078			
Laterality						
Left	Ref					
Other	8.04	4.4–14.69	<0.001			
Right	1.06	0.92–1.22	0.403			
Fuhrman.grade						
I	Ref			Ref		
II	0.98	0.75–1.29	0.898	0.84	0.64–1.09	0.19
III	1.1	0.83–1.45	0.521	1.12	0.85–1.46	0.43
IV	1.16	0.84–1.6	0.358	1.6	1.18–2.18	0.003
T.Stage						
T1	Ref			Ref		
T2	3.28	2.43–4.43	<0.001	0.71	0.53–0.97	0.029
T3	1.88	1.44–2.46	<0.001	0.87	0.67–1.13	0.31
T4	3.04	2.06–4.49	<0.001	1	0.72–1.38	0.99
TX	6.73	4.95–9.16	<0.001	0.75	0.55–1.03	0.072
N.Stage						
N0	Ref			Ref		
N1	3.23	2.63–3.97	<0.001	1.12	0.92–1.36	0.25
NX	2.85	2.13–3.81	<0.001	1.28	1.01–1.63	0.042
Tumor.size						
≤4	Ref			Ref		
≥10	1.05	0.77–1.43	0.755	1.44	1.05–1.97	0.022
4–7	2.47	1.97–3.09	<0.001	1.26	0.98–1.61	0.067
7–10	1.45	1.07–1.95	0.015	1.48	1.1–2	0.009
Invasion.beyond.capsule						
Lateral and medial invasion	Ref			Ref		
Lateral invasion	1.14	0.8–1.62	0.461	0.92	0.7–1.21	0.56
Medial invasion	1.17	0.81–1.71	0.398	0.99	0.75–1.3	0.92
Not present	0.85	0.59–1.22	0.371	1.04	0.81–1.35	0.75
Brain.metastasis						
No	Ref			Ref		
Yes	2.31	1.74–3.09	<0.001	1.67	1.34–2.08	<0.001
Liver.metastasis						
No	Ref			Ref		
Yes	1.77	1.35–2.32	<0.001	1.22	0.98–1.52	0.068
Lung.metastasis						
No	Ref			Ref		
Yes	5	4.16–6.01	<0.001	1.21	1.03–1.43	0.022
Surgery						
None				Ref		
Local tumor excision/destruction				1.66	0.73–3.78	0.23
Nephrectomy, NOS				0.46	0.2–1.07	0.07
Partial nephrectomy				0.29	0.18–0.47	<0.001
Radical nephrectomy				0.4	0.33–0.48	<0.001
Surgery, NOS				0.65	0.42–0.99	0.046
Lymph.node.removal						
No				Ref		
Yes				1.03	0.83–1.29	0.76

### Development and Validation of Nomograms

**Figure 3:**
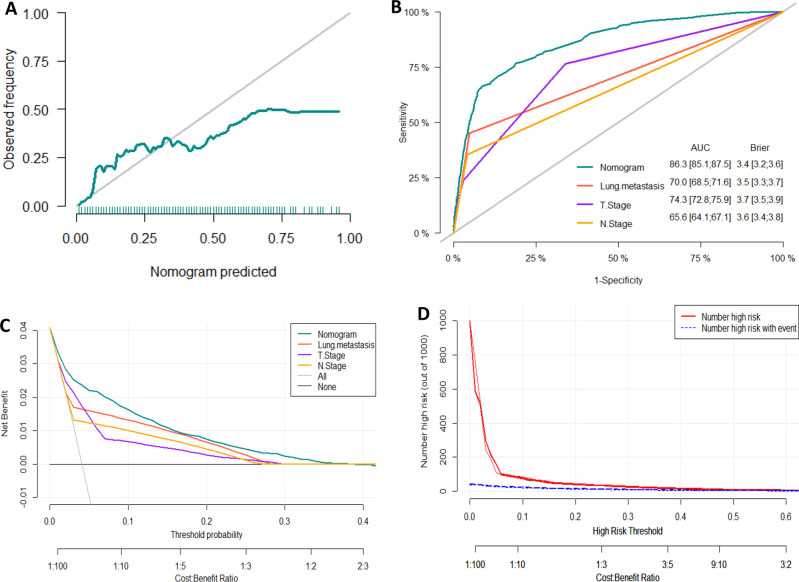
Evaluation of the nomogram for risk assessment of bone metastasis in the training dataset. (**A**) Calibration plot. (**B**) The receiver operating characteristic curves (ROC) of Nomogram, Lung metastasis, T stage and N stage. (**C**) Decision curve analysis (DCA) of Nomogram, Lung metastasis, T stage and N stage. (**D**) Clinical impact Curve.

**Figure 4:**
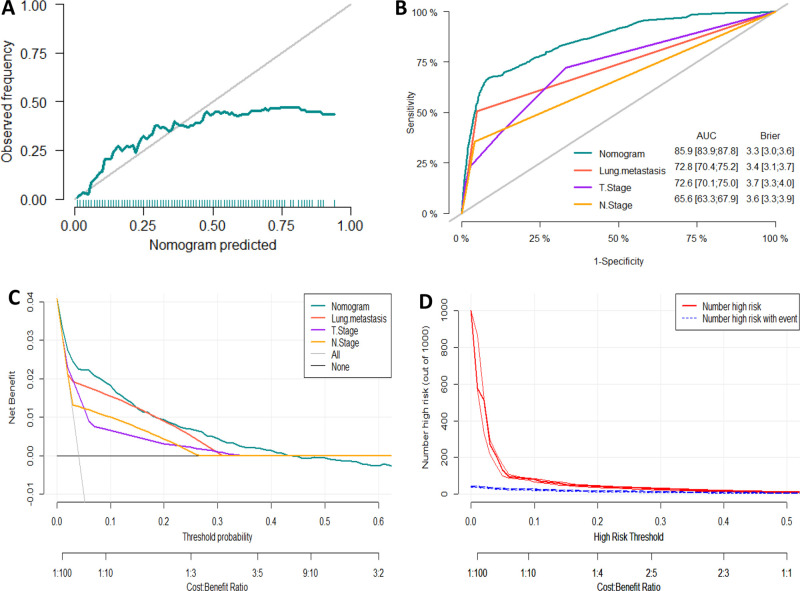
Evaluation of the nomogram for risk assessment of bone metastasis in the testing dataset. (**A**) Calibration plot. (**B**) The receiver operating characteristic curves (ROC) of Nomogram, Lung metastasis, T stage and N stage. (**C**) Decision curve analysis (DCA) of Nomogram, Lung metastasis, T stage and N stage. (**D**) Clinical impact Curve.

**Figure 5:**
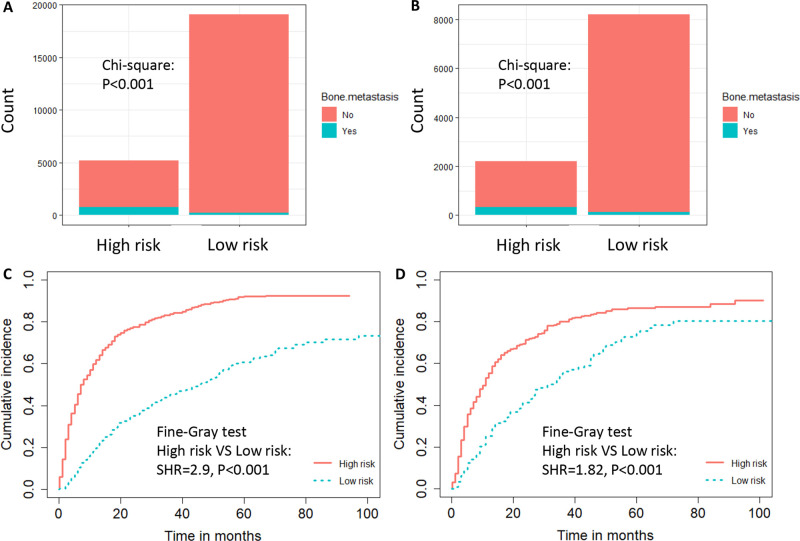
The risk-classification performance of the diagnostic nomogram in the training dataset (**A**) and testing dataset (**B**). Cumulative incidence function (CIF) curves with the *P* value of Fine-gray test for the training dataset (**C**) and testing dataset (**D**) in the prognostic cohort.

We developed a diagnostic nomogram to assess the risk of BM in ccRCC patients based on multivariate logistic regression analysis (**Figure 2B**). In our nomogram, the contribution of variables to the final probability consisted of their respective line lengths and corresponding fractions. Individual scores were obtained for the different patients. The probability of developing BM was determined using the total score obtained by calculating the scores of each variable. The calibration curves exhibited good consistency between the observation and prediction results (**Figures 3A, 4A**). In both cohorts, the nomogram showed better discriminant ability than individual predictive variables, with areas under the curves of 0.863 and 0.859, respectively (**Figures 3B, 4B**). The cutoff value of the ROC curve in the training set was 85.6. According to the cutoff value, ccRCC patients were classified into a low-risk group (total score <85.6) and high-risk group (total score ≥85.6). It was obvious that patients in the high-risk group were at a greater risk of BM in the two cohorts (*P* < 0.001) (**Figures 5A,B**). Additionally, the analysis of the decision curve analysis demonstrated the superior clinical practice value of the nomogram: when the incidence rate was >30%, the event probability was completely consistent with on the model. (**Figures 3C,D, 4C,D**). We developed a web-based online calculator for BM risk prediction in patients with ccRCC. By clicking the option of each variable on the website, the corresponding BM risk score can be easily obtained (https://vincent–267y.shinyapps.io/Online_nomogram_for_ccRCC_bmrisk/).

### Prognostic Factors for ccRCC-BM and Relative Importance

A total of 1,415 patients with eligible ccRCC-BM were included in the study for prognostic factor analysis (**Table 1**). We identified 568 (40.1%) patients that underwent radical nephrectomy, 190 (13.4%) underwent lymphadenectomy, 864 (61.1%) received radiotherapy, and 840 (59.4%) received chemotherapy (Supplementary Table S7). There were no significant differences between the baseline data from the training and testing datasets. Univariate and multivariate competing risk analysis showed that T stage, N stage, Fuhrman grade, tumor size, lung metastasis, brain metastasis, and surgery could independently predict CSS (**Table 2** and Supplementary Table S6). Surgical management was the most important independent prognostic factor, followed by the Fuhrman grade, brain metastasis, etc. (**Figure 2C**).

### Establishment and Validation of a Prognostic Nomogram

**Figure 6:**
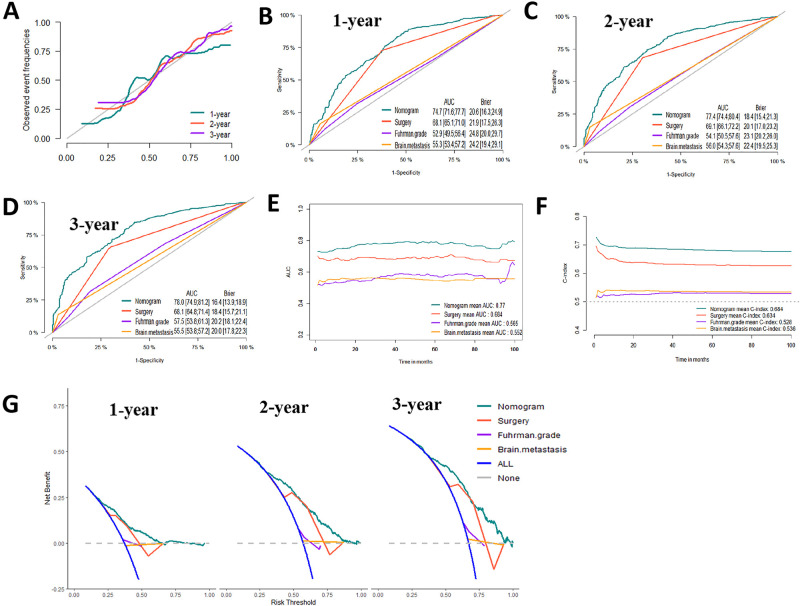
Evaluation of the competing-risk nomogram on training dataset for cancer-specific survival (CSS). (**A**) Calibration plot of the 1-,2- and 3-year CSS nomogram. (**B**) 1-year, (**C**) 2-year and (**D**) 3-year receiver operating characteristic curves (ROC) of Nomogram, Surgery management, Fuhrman Grade and Brain metastasis. The continuous AUCs (**E**) and C-index (**F**) of Nomogram, Surgery management, Fuhrman Grade and Brain metastasis for CSS. (**G**) Decision curve analysis (DCA) of Nomogram, Surgery management, Fuhrman Grade and Brain metastasis.

**Figure 7:**
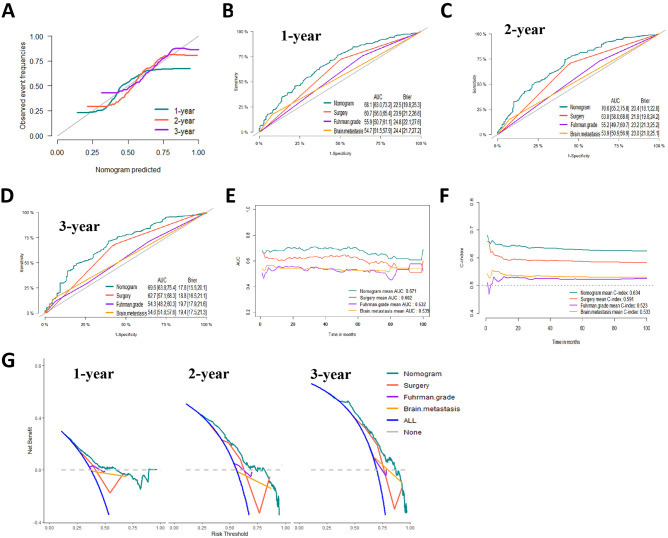
Evaluation of the competing-risk nomogram on testing dataset for cancer-specific survival (CSS). (**A**) Calibration plot of the 1-,2- and 3-year CSS nomogram. (**B**) 1-year, (**C**) 2-year and (**D**) 3-year receiver operating characteristic curves (ROC) of Nomogram, Surgery management, Fuhrman Grade and Brain metastasis. The continuous AUCs (**E**) and C-index (**F**) of Nomogram, Surgery management, Fuhrman Grade and Brain metastasis for CSS. (**G**) Decision curve analysis (DCA) of Nomogram, Surgery management, Fuhrman Grade and Brain metastasis.

Based on the prognostic factors identified by multivariate competing risk analysis, a nomogram was established to predict the 1-, 2-, and 3- year CSS in patients with ccRCC-BM (**Figure 2D**). The nomogram suggested excellent agreement between probability and actual observation of CSS at 1, 2, and 3 years, as illustrated in the calibration curves (**Figures 6A, 7A**). In the training cohort, the areas under the curves of 1, 2, and 3 years were 0.747, 0.774 and 0.780, respectively, while the areas under the curves of 1, 2, and 3 years in the testing cohort were 0.681, 0.706, and 0.696, respectively. Furthermore, the nomogram showed better discrimination than the other independent single predictors (**Figures 6B–D and 7B–D**). In addition, we compared the continuous trend of the predictive performance of the nomogram and the independent variables. As shown in **Figures 6E,F, 7E,F**, the area under the curve and C-index of the nomogram were higher than those of other variables in the training dataset and testing dataset over time. Decision curve analysis showed that the nomogram had excellent efficiency in predicting CSS in ccRCC-BM patients and had better clinical net benefit than surgery, Fuhrman grade, and brain metastasis (**Figures 6G, 7G**). According to the results of the X-tile calculation, the best cutoff value was 92.2. Scores greater than 92.2 were considered high risk, while scores less than 92.2 were considered low risk. Patients assigned to the high-risk group had worse prognoses in the training and testing cohorts (**Figures 5C,D**). Clinicians can successfully distinguish risk groups when a nomogram is a predictive tool. We also developed a Web-based online calculator for the prognostic prediction of ccRCC-BM in patients with CSS. (https://vincent–267y.shinyapps.io/Online_nomogram_for_ccRCC_prediction_CPRCSS/).

## Discussion

In ccRCC patients, bone metastases can cause skeletal-related adverse events, lead to substantial morbidity, and often predict poor outcomes (18). Therefore, it is necessary to identify the risk and prognostic factors of bone metastasis in patients with ccRCC to improve their survival. In this study, logistic regression analyses were used to analyze the risk factors associated with BM, and competing risk analyses were used to evaluate the prognosis of ccRCC-BM patients. In addition, we constructed diagnostic and prognostic nomograms, which may help clinicians detect BM promptly and conduct clinical evaluation and intervention at an early stage. In addition, we have developed online calculators for clinicians to assess the risk and prognosis of patients with ccRCC-BM.

In our study, elderly patients, tumor size, laterality, T stage, N stage, brain metastasis, liver metastasis, and lung metastasis were important predictors of BM in ccRCC patients. Younger patients with ccRCC have a lower risk of developing BM at initial diagnosis, supporting evidence that older RCC patients have a higher risk of metastases (19, 20). Some previous studies have shown that tumor size is significantly correlated with the risk of metastasis in patients with ccRCC, with a negligible risk of metastasis in patients with tumors smaller than 3 cm (21). Our study confirmed the observation that patients with ccRCC with tumor diameter of 4–10 cm had an increased risk of BM in multiple logistic regression analysis. However, it should be noted that there is no difference in the risk of developing BM between patients with tumor diameters >10 cm and those with tumor diameters <4 cm. This phenomenon needs to be clarified in future studies. The effect of laterality on disease outcomes in renal cell carcinoma remains unclear. More collateral circulation in the left renal vein may lead to increased metastasis (22). Notably, in this study, the risk of developing BM on the left was similar to that on the right, while patients with bilateral or other types had a higher risk of BM, which was consistent with a previous study (23). A previous report indicated that T and N stages are important predictors of distant metastasis in patients with ccRCC (24). In our study, higher grades of T and N staging were associated with a higher risk of BM at diagnosis. Lung, brain, and liver metastases are significantly associated with BM in patients with ccRCC. Several studies have described similar phenomena (14, 15, 23). Our study showed that ccRCC patients with lung, brain, and liver metastases are more likely to develop bone metastases. One possible hypothesis is that tumor cells have escaped in patients with lung/brain/liver metastases and their subsequent hematogenous and lymphatic spread contributes to an increased risk of bone metastases (25), but the complex mechanisms still need to be further investigated.

There are limited studies on prognostic factors in patients with ccRCC with BM. Huang et al. determined that shorter time to bone metastasis, older age, multiple organ metastasis, and lack of CA-IX expression were associated with a poor prognosis in patients with ccRCC-BM. However, it did not describe prognostic factors in patients with ccRCC-BM at a large population level. Our study used competing risk analysis to effectively eliminate the effects of other causes of death on cancer-specific mortality in patients with ccRCC-BM. Competing risk analysis showed better performance in predicting disease-specific outcomes and can better estimate the prognosis of patients and help clinicians make appropriate treatment decisions (26). According to the results of competing risk regression models, Fuhrman grade IV was associated with a poor prognosis. Fuhrman grade is an important prognostic factor for ccRCC, as confirmed in several studies (27, 28).

Many previous studies have found that the T and N stages play an important role in predicting survival outcomes in patients with RCC with bone metastases. Higher T and N stages are associated with a worse prognosis (14, 15). Interestingly, T2 patients had a better prognosis than T1 patients in our study. Since most T and N stages in the SEER database are clinical stages based on imaging examinations, this conclusion may not be accurate (29). In addition, we found no significant differences in the prognosis of N1 patients compared to N0 patients. In contrast, NX patients had a worse prognosis, which may have little significance in practical clinical applications. Because the N stage of patients with NX is unknown, more detailed analyses are necessary for the future. We observed that brain metastases and lung metastases suggested a poor prognosis in patients with ccRCC-BM, which was consistent with the findings of Xue et al. Among patients with mRCC, those with multiple metastases had a worse outcome than single metastases, and the prognosis worsens as the number of metastases increases (24). Similar phenomena have also been observed in other malignant tumors (30, 31).

Zhi et al. found that a larger tumor size was associated with a higher risk of lymph node metastasis and a poorer prognosis of ccRCC, with overall survival and CSS gradually decreasing as tumor size increased (32). Our study found that patients with tumors >7 cm had a worse prognosis. Considering that tumor size is an independent indicator of the risk and prognosis of bone metastases in ccRCC patients, more attention should be paid to patients with larger tumors when guiding clinical decision-making. Even in the era of targeted therapy, cytoreductive nephrectomy remains an important treatment for mRCC. A previous study showed that radical nephrectomy can improve the survival outcomes of patients with ccRCC-BM (33). Our study showed that ccRCC patients who underwent RN or PN had a better prognosis.

This study had several limitations. First, the prediction model was constructed based on the SEER database, which did not contain key clinical information, such as detailed information on patients’ systemic treatment, molecular biomarkers of bone lesions, and laboratory indicators. Second, skeletal-related events are an important prognostic factor for BM, and the SEER database does not contain this information. Third, missing values were processed using multiple imputation techniques, which may reduce the model’s performance. In addition, the constructed nomograms are based on clinical information from patients in the SEER database, which comprises approximately 30% of the total US population. Therefore, further validation using data from other country would be helpful to improve the model’s generalization ability and expand the population for whom the nomogram is applicable. Finally, considering that this was a retrospective study and selection bias may have occurred during the study, it is necessary to further verify the accuracy of our nomograms through more clinical trials or prospective cohort studies.

## Conclusions

We retrospectively analyzed the clinical characteristics of the risk of BM in patients with ccRCC and the prognostic factors of patients with BM based on the SEER database. Our study determined that age, tumor size, laterality, T stage, N stage, brain metastasis, liver metastasis, and lung metastasis were risk factors for BM in ccRCC patients. T stage, N stage, Fuhrman grade, tumor size, lung metastasis, brain metastasis, and surgery were independent prognostic factors for patients with ccRCC-BM. The two established nomograms showed excellent calibration, discrimination, and clinical utility. Nomograms and web-based online calculators are expected to become effective and precise tools for clinicians to improve cancer management.

## Data Availability

The original contributions presented in the study are included in the article/supplementary material, further inquiries can be directed to the corresponding author/s.
